# Quality of life before and during the COVID-19 pandemic for people undergoing hip, knee and shoulder arthroplasty—nationwide results from the Australian Orthopaedic Association National Joint Replacement Registry

**DOI:** 10.1007/s00264-026-06778-3

**Published:** 2026-03-24

**Authors:** Stephen D Gill, Richard S Page, Qunyan Xu, Ian A Harris, David Gill, Ilana N Ackerman

**Affiliations:** 1 School of Medicine, Deakin University, Waurn Ponds, Australia; 2 South Australian Health and Medical Research Institute, Adelaide, Australia; 3 Southwest Sydney Campuses, School of Clinical Medicine, UNSW Medicine & Health, UNSW Sydney, Sydney, Australia; 4 Australian Orthopaedic Association National Joint Replacement Registry, Adelaide, Australia; 5 School of Public Health and Preventive Medicine, Monash University, Melbourne, Australia; 6 Barwon Centre for Orthopaedic Research and Education (B-CORE), St John of God Hospital and Barwon Health, Geelong, Australia

**Keywords:** Quality of life, Arthroplasty, COVID-19, Pandemic, Hip, Knee, Shoulder

## Abstract

**Background:**

The COVID-19 pandemic produced a substantial reduction in arthroplasties, which could have affected patient quality of life. This study investigated quality of life in Australians undergoing elective arthroplasty before and during the COVID-19 pandemic.

**Methods:**

Using data from the Australian Orthopaedic Association National Joint Replacement Registry, quality of life was assessed in patients before and six months after primary total hip arthroplasty (THA), total knee arthroplasty (TKA) and reverse total shoulder arthroplasty (RTSA) using the EQ-5D-5L instrument. Patients with an EQ-5D Utility score less than zero were considered to have a quality of life worse than dead. Secondary outcomes included Oxford Scores, joint-specific pain, patient perceived change, responder status and patient satisfaction. Quality of life was compared before (1 July 2018–10 March 2020) and during the pandemic (11 March 2020–10 March 2023) using linear or logistic regression models.

**Results:**

The analysis included preoperative data for more than 24,000 THA patients, 20,000 TKA patients and 1,100 RTSA patients. Compared to the pre-pandemic period, preoperative and postoperative quality of life significantly reduced during the pandemic for THA and TKA, but not by a meaningful amount (adjusted mean difference ≤ 0.03 points, *p* < 0.001). The likelihood of having quality of life worse than dead increased during the pandemic but was only significant for preoperative THA (ORs 1.24 to 1.40, *p* < 0.02). For secondary outcomes, joint-specific scores deteriorated, and joint pain increased to a small degree during the pandemic for THA and TKA (*p* < 0.05), but not for RTSA. The proportion of THA and TKA patients satisfied with their surgery outcome increased modestly during the pandemic by approximately five percentage points, compared to pre-pandemic.

**Conclusion:**

The COVID-19 pandemic was not associated with a clinically meaningful deterioration in pre- or post-operative quality of life, on average, for patients undergoing THA, TKA or RTSA in Australia.

## Introduction

The COVID-19 pandemic produced a dramatic reduction in elective arthroplasty throughout the world. In Australia, Federal Government directives prioritised emergency and urgent surgery, which saw an immediate 83.7% reduction in primary arthroplasty in the ensuing month [1]. Although elective surgery gradually resumed over subsequent months, resumption rates varied throughout the country in line with local pandemic policies. Public hospitals experienced the greatest reduction in arthroplasty volume, down 22.6% from the pre-pandemic year [1].

It is well established that people awaiting arthroplasty experience considerable pain and disability, which is prolonged by delays in surgery. Furthermore, at a population-level, the COVID-19 pandemic was associated with reduced quality of life [2]. Hence, delays in surgery, combined with the direct impact of the pandemic on quality of life, makes it reasonable to expect that those awaiting arthroplasty would experience reduced quality of life during the pandemic. In the UK, Clement et al. observed a substantial reduction in quality of life in people awaiting arthroplasty during the pandemic [3]. Almost one-quarter and one-third of people awaiting total hip arthroplasty (THA) or total knee arthroplasty (TKA), respectively, reported quality of life scores that equated to a state ‘worse than dead’. These levels were almost double those observed prior to the pandemic [4].

The impact of the COVID-19 pandemic on Australians awaiting arthroplasty is currently unknown. Further deterioration in quality of life, similar to those identified by Clement et al. [5], would signal a need for targeted interventions to address this health care priority. The aim of this study is to investigate quality of life in Australians undergoing elective arthroplasty during the COVID-19 pandemic, as compared to before the pandemic. Specifically, we compared preoperative and postoperative quality of life in patients awaiting THA, TKA or reverse total shoulder arthroplasty (RTSA) in the years before (2018–2020) and during the pandemic (2020–2023). Secondary aims were to compare, before and during the pandemic, 1) pain and function scores, 2) the number of people whose quality of life was considered ‘worse than dead’, and 3) satisfaction with surgery.

## Methods

### Study design

The study was a registry-based nationwide cohort study.

### Patients

Eligible patients were those undergoing primary conventional THA, TKA or RTSA for osteoarthritis in Australia between 1 July 2018 and 10 March 2023. Anatomical TSA were excluded due to insufficient patient numbers (71% of primary TSR procedures in Australia in 2023 were RTSA [6]). Patients undergoing partial or revision surgery were excluded.

### Data collection

All demographic, procedural and patient-reported outcomes (PROMs) data were collected by the Australian Orthopaedic Association National Joint Replacement Registry (AOANJRR). The AOANJRR monitors outcomes following arthroplasty for almost all Australian patients, with revision surgery as the primary endpoint. In July 2018, the registry commenced collecting PROMs. An initial pilot study included 44 hospitals, which comprised public and private hospitals from all States and one Territory. Subsequently, the PROMs program was expanded and now includes 340 Australian hospitals. PROMs are collected electronically from consenting patients preoperatively and at six months postoperatively. The registry also collects information regarding patient age, gender, BMI category, American Society of Anesthesiologists (ASA) score [7], and hospital system (public or private hospital).

### Outcomes

For this study, the EQ-5D-5L was the primary outcome measure, which is a commonly used generic health-related quality of life instrument that includes five domains: pain/discomfort, mobility, self-care, usual activities and anxiety/depression [8]. The EQ-5D Utility score summarises the five domains into a single score to indicate quality of life based on the population’s values and preferences for particular health states. A utility score of 1.0 represents full health, and a score less than zero represents quality of life considered to be worse than dead (WTD). Patients with a combination of extreme pain, poor mobility, inability to perform self-care or perform usual activities, and severe anxiety/depression, have health states that are often valued by the population as intolerable, leading to utility scores less than zero. For the current study, utility scores were derived from recently updated value sets for the Australian population [9]. Norman et al. used discrete choice experiments in which Australian adults chose the best and worst option from three health states. Being dead was the third option. Consequently, participants indicated which health states were considered worse than dead (i.e. when being ‘dead’ was selected as the best option, the remaining options were consider ‘worse than dead’) [9]. Using this value set, the lowest possible score (indicating poorest health) is −0.30. Utility scores were categorised into two groups (EQ Utility Category): WTD and not WTD. Secondary outcomes included:

1. EQ VAS: generic measure of overall patient-rated health [8]. Scores range from 0 to 100, with higher scores representing better health.
2. Oxford Hip, Knee and Shoulder Scores: 12-item joint-specific measures of pain and function [10, 11]. Scores range from 0 to 48, with higher scores representing better hip-, knee- or shoulder-specific health, respectively.
3. Joint-specific pain: 0 to 10 rating scale with 10 being the worst hip, knee or shoulder pain imaginable.
4. Perceived change since before surgery (collected post-operatively only): 5-point Likert scale including “much better” and “a little better” (classified as ‘improved’), and “about the same”, “a little worse” and “much worse” (classified as ‘not improved’).
5. Patient satisfaction after surgery (collected post-operatively only): 5-point Likert scale including “very satisfied” and “satisfied” (classified as ‘satisfied’), and “neutral”, “dissatisfied” and “very dissatisfied” (classified as ‘unsatisfied’).
6. Responder: For this study, a patient was considered a ‘responder’ if their change in Oxford Hip/Knee/Shoulder Score between the preoperative and postoperative was greater than or equal to the published minimal important change of 8 points for THA [12], 7 points for TKA [12], and 9 points for RTSA [13].

### Data analysis

The pre-pandemic period was defined as prior to the World Health Organization’s pandemic declaration on 11 March 2020. Three consecutive time periods during the pandemic were each compared to the control period:

- Pre-pandemic: 1 July 2018 to 10 March 2020
- Pandemic Year 1: 11 March 2020 to 10 March 2021
- Pandemic Year 2: 11 March 2021 to 10 March 2022
- Pandemic Year 3: 11 March 2022 to 10 March 2023

Three consecutive periods were chosen to allow evaluation of the effects of the pandemic over time. Data for the THA, TKA and RTSA cohorts were analysed separately. Six covariates were considered simultaneously in the analysis and adjusted in the models as competing exposures: age (< 65 years vs ≥ 65 years), gender (male or female), BMI category (normal and underweight(< 25)/overweight(25 < 30)/Obese Class I(30 < 35)/Obese Class II(35 < 40)/Obese Class III(≥ 40), American Society of Anesthesiologists (ASA) score (1-V) [7], hospital system (publicly or privately funded hospital) and location of hospital (State of Victoria versus other States/Territories).

The effects of pandemic year (exposure) on outcomes were estimated by fitting a separate regression model for each PROM. Linear regression was fitted for continuous PROMs (EQ-5D Utility score, EQ-VAS, Oxford Hip/Knee/Shoulder Scores, joint-specific pain), where the mean difference was used as the effect measure. Logistic regression was fitted for binary PROMs (perceived change, satisfaction, responder status and EQ Utility Category), where the odds ratio (OR) was the effect measure.

In addition to the primary analysis, pairwise comparisons between each pandemic year and the pre-pandemic period (e.g., pandemic year 1 vs pre-pandemic) were conducted. The estimated main effects of each pandemic year relative to the pre-pandemic period were reported as either mean difference (95% CI) or OR (95% CI) for continuous or binary outcomes, respectively. In all analyses, the main effects of pandemic year were first estimated without considering other covariates (unadjusted model), followed by a model that controlled for the other six covariates (adjusted model).

The significance level was 0.05 for all comparisons. As multiple comparisons increase the risk of false positive findings, conclusions were based on overall patterns observed across outcomes to reduce the risk of Type 1 errors. For example, if all PROMs demonstrated statistically significant findings for a particular comparison, then a true effect was considered likely.

To assist with interpreting results, mean between-group differences from pairwise comparisons of continuous outcomes were descriptively compared to published minimum clinically important differences (MCID) for each measure. The MCID for between-groups comparisons for the OHS and OKS is 5 points [14]. When MCID was not available from the literature, we used MCIC (within-group change) as a conservative guide. On this basis, an important change was assumed to be 9 points for the OSS [13], 0.2 utility units for the EQ-5D-5L [15], 5 points for the EQ VAS [16], and 2 points for joint-specific pain [17].

### Ethical declarations

The Australian Orthopaedic Association National Joint Replacement Registry (AOANJRR) is a national Quality Improvement Program to audit the outcome of joint replacement in Australia. It is an initiative of the Australian Orthopaedic Association (AOA) and is permanently funded by the Commonwealth Government. It was established in 1999 and is listed as a Declared Quality Assurance Activity under Section 124X of the Health Insurance Act 1973 (QAA 3/2017). The study has been conducted in accordance with the Declaration of Helsinki. All patients consented to participation in the PROMs program.

## Results

A summary of patient characteristics is provided in Table 1.

**Table 1:** Patient characteristics for hip, knee and shoulder arthroplasty

		Pre-pandemic	Pandemic Year 1	Pandemic Year 2	Pandemic Year 3
**TOTAL HIP ARTHROPLASTY (THA)**					
Age (years)	Mean ± SD	67 ± 10.6	66.6 ± 10.6	66.3 ± 10.5	66.9 ± 10.4
Gender	Female	3,068 (54.6%)	1,809 (52%)	3,637 (53.3%)	5,871 (54.3%)
	Male	2,548 (45.4%)	1,668 (48%)	3,193 (46.7%)	4,933 (45.7%)
BMI Category combined^1^	Underweight or Normal	1,169 (21%)	700 (20.3%)	1,398 (20.6%)	2,203 (20.5%)
	Overweight	1,957 (35.2%)	1,224 (35.6%)	2,465 (36.3%)	3,930 (36.6%)
	Obese Class 1	1,450 (26.1%)	929 (27%)	1,765 (26%)	2,813 (26.2%)
	Obese Class 2	641 (11.5%)	399 (11.6%)	768 (11.3%)	1,205 (11.2%)
	Obese Class 3	339 (6.1%)	190 (5.5%)	387 (5.7%)	583 (5.4%)
ASA Score^2^	1	456 (8.1%)	280 (8.1%)	562 (8.2%)	731 (6.8%)
	2	3,136 (55.9%)	1,939 (55.9%)	3,849 (56.4%)	5,790 (53.7%)
	3	1,954 (34.8%)	1,213 (34.9%)	2,354 (34.5%)	4,145 (38.4%)
	4	62 (1.1%)	39 (1.1%)	59 (0.9%)	119 (1.1%)
	5				3 (0%)
Hospital System	Private	2,634 (46.9%)	1,764 (50.7%)	4,898 (71.7%)	8,213 (76%)
	Public	2,982 (53.1%)	1,713 (49.3%)	1,932 (28.3%)	2,591 (24%)
TOTAL (THA)		5,616	3,477	6,830	10,804
**TOTAL KNEE ARTHROPLASTY (TKA)**					
Age (years)	Mean ± SD	67.6 ± 8.8	67.5 ± 9	67.2 ± 8.5	67.7 ± 8.5
Gender	Female	4,924 (56.4%)	3,068 (55.2%)	5,620 (53.9%)	9,061 (54%)
	Male	3,812 (43.6%)	2,488 (44.8%)	4,802 (46.1%)	7,721 (46%)
BMI Category combined^3^	Underweight or Normal	860 (9.9%)	524 (9.5%)	1,091 (10.5%)	1,793 (10.7%)
	Overweight	2,525 (29.2%)	1,738 (31.6%)	3,205 (30.9%)	5,266 (31.5%)
	Obese Class 1	2,653 (30.7%)	1,633 (29.7%)	3,241 (31.3%)	5,088 (30.5%)
	Obese Class 2	1,537 (17.8%)	956 (17.4%)	1,739 (16.8%)	2,847 (17%)
	Obese Class 3	1,071 (12.4%)	656 (11.9%)	1,082 (10.4%)	1,714 (10.3%)
ASA Score^4^	1	419 (4.8%)	267 (4.8%)	514 (4.9%)	794 (4.7%)
	2	4,679 (53.7%)	2,924 (52.9%)	5,693 (54.7%)	8,804 (52.5%)
	3	3,539 (40.6%)	2,287 (41.4%)	4,122 (39.6%)	7,022 (41.9%)
	4	79 (0.9%)	49 (0.9%)	87 (0.8%)	152 (0.9%)
Hospital System	Private	4,049 (46.3%)	2,920 (52.6%)	7,539 (72.3%)	13,024 (77.6%)
	Public	4,687 (53.7%)	2,636 (47.4%)	2,883 (27.7%)	3,758 (22.4%)
TOTAL (TKA)		8,736	5,556	10,422	16,782
**REVERSE TOTAL SHOULDER ARTHROPLASTY (RTSA)**					
Age (years)	Mean ± SD	72.6 ± 7.4	72.8 ± 7.2	70.7 ± 7.4	72.4 ± 7.1
Gender	Female	147 (64.2%)	80 (67.2%)	207 (63.7%)	350 (58.6%)
	Male	82 (35.8%)	39 (32.8%)	118 (36.3%)	247 (41.4%)
BMI Category combined^5^	Underweight or Normal	35 (15.6%)	16 (13.6%)	40 (12.4%)	73 (12.4%)
	Overweight	71 (31.6%)	31 (26.3%)	112 (34.7%)	197 (33.3%)
	Obese Class 1	57 (25.3%)	33 (28%)	91 (28.2%)	176 (29.8%)
	Obese Class 2	39 (17.3%)	28 (23.7%)	49 (15.2%)	87 (14.7%)
	Obese Class 3	23 (10.2%)	10 (8.5%)	31 (9.6%)	58 (9.8%)
ASA Score^6^	1	2 (0.9%)	3 (2.5%)	9 (2.8%)	15 (2.5%)
	2	100 (43.9%)	54 (45.4%)	130 (40%)	233 (39.2%)
	3	122 (53.5%)	58 (48.7%)	178 (54.8%)	332 (55.8%)
	4	4 (1.8%)	4 (3.4%)	8 (2.5%)	15 (2.5%)
Hospital System	Private	124 (54.1%)	65 (54.6%)	234 (72%)	472 (79.1%)
	Public	105 (45.9%)	54 (45.4%)	91 (28%)	125 (20.9%)
TOTAL (RTSA)		229	119	325	597

*SD* standard deviation, *BMI* body mass index (kg/m^2^), *ASA* American Society of Anesthesiologists

^1^Excludes 212 procedures with unknown BMI category combined; ^2^Excludes 36 procedures with unknown ASA Score; ^3^Excludes 277 procedures with unknown BMI category combined; ^4^Excludes 65 procedures with unknown ASA Score; ^5^Excludes 13 procedures with unknown BMI category combined; ^6^Excludes 3 procedures with unknown ASA Score

For THA, over 24,000 and 20,000 patients were included in the preoperative analysis and postoperative analysis for most outcomes, respectively; for TKA, over 37,000 and 31,000 were included, and for RTSA, over 1,100 and 900 patients were included. Patients receiving surgery in private hospitals represented 65.5%, 66.3% and 70.5% of the THA, TKA and RTSA cohorts, respectively.

Mean EQ-5D Utility scores improved substantially following surgery for all three cohorts, typically from 0.6 preoperatively to 0.9 postoperatively (Table 2). Preoperatively, the proportion of patients with quality of life worse than dead was low (4.2% to 5.8% for THA, 1.8% to 2.3% for TKA, and 1.2% to 1.9% for RTSA), and decreased further postoperatively (≤ 0.5% for THA and TKA, and ≤ 1.2% for RTSA).

**Table 2:** Patient reported outcomes for total hip, knee and shoulder arthroplasty

		Pre-pandemic	Pandemic Year 1	Pandemic Year 2	Pandemic Year 3
**TOTAL HIP ARTHROPLASTY(THA)**					
Preop EQ Utility Score	Mean ± SD	0.6 ± 0.3	0.5 ± 0.3	0.6 ± 0.3	0.6 ± 0.3
Preop EQ VAS Score	Mean ± SD	67.1 ± 20	65.9 ± 20.3	66.3 ± 20.2	65.9 ± 19.9
Preop OHS	Mean ± SD	20.6 ± 8.8	20.2 ± 9	21.3 ± 9.1	21.2 ± 9.1
Preop Joint Pain Score	Mean ± SD	6.9 ± 2.1	7 ± 2	6.8 ± 2.1	6.8 ± 2.1
Postop EQ Utility Score	Mean ± SD	0.9 ± 0.2	0.9 ± 0.2	0.9 ± 0.2	0.9 ± 0.2
Postop EQ VAS Score	Mean ± SD	81.1 ± 16	79.7 ± 16.5	81.1 ± 15.7	81.4 ± 15.2
Postop OHS	Mean ± SD	41.6 ± 7.3	40.6 ± 7.7	41.6 ± 7.1	41.7 ± 7
Postop Joint Pain Score	Mean ± SD	1.4 ± 2.2	1.6 ± 2.3	1.5 ± 2.1	1.5 ± 2.2
Preop EQ Utility Category	<0 ‘worse than dead’	254 (4.7%)	192 (5.8%)	266 (4.2%)	435 (4.5%)
	≥0	5,098 (95.3%)	3,121 (94.2%)	6,096 (95.8%)	9,269 (95.5%)
Postop EQ Utility Category	<0 ‘worse than dead’	21 (0.5%)	15 (0.5%)	21 (0.4%)	43 (0.5%)
	≥0	4,511 (99.5%)	2,855 (99.5%)	4,967 (99.6%)	8,398 (99.5%)
Patient Perceived Change	Improved	4,357 (96.8%)	2,719 (95.7%)	4,767 (96.7%)	8,111 (97%)
	Not-improved	143 (3.2%)	123 (4.3%)	164 (3.3%)	249 (3%)
Patient Satisfaction	Satisfied	3,914 (87%)	2,588 (91.1%)	4,595 (93.2%)	7,812 (93.4%)
	Not-satisfied	587 (13%)	254 (8.9%)	337 (6.8%)	551 (6.6%)
Responder	Responder	3,821 (90.8%)	2,405 (90%)	4,021 (90%)	6,528 (89.6%)
	Non-responder	385 (9.2%)	267 (10%)	446 (10%)	758 (10.4%)
TOTAL (THA)		5,616	3,477	6,830	10,804
**TOTAL KNEE ARTHROPLASTY (TKA)**					
Preop EQ Utility Score	Mean ± SD	0.6 ± 0.3	0.6 ± 0.3	0.7 ± 0.3	0.7 ± 0.3
Preop EQ VAS Score	Mean ± SD	69.2 ± 18.5	70 ± 18.8	70.3 ± 18.1	70.4 ± 17.9
Preop OKS	Mean ± SD	22 ± 8.3	22.3 ± 8.4	23 ± 8.4	23.3 ± 8.4
Preop Joint Pain Score	Mean ± SD	6.7 ± 2.1	6.7 ± 2.1	6.5 ± 2.1	6.5 ± 2.1
Postop EQ Utility Score	Mean ± SD	0.9 ± 0.2	0.9 ± 0.2	0.9 ± 0.2	0.9 ± 0.2
Postop EQ VAS Score	Mean ± SD	79.6 ± 15.9	79.3 ± 15.9	80.3 ± 15	80.3 ± 15.3
Postop OKS	Mean ± SD	37.6 ± 8	37.5 ± 8	37.8 ± 7.7	38 ± 7.7
Postop Joint Pain Score	Mean ± SD	2.3 ± 2.4	2.4 ± 2.4	2.4 ± 2.3	2.4 ± 2.4
Preop EQ Utility Category	<0 ‘worse than dead’	189 (2.3%)	116 (2.2%)	181 (1.9%)	274 (1.8%)
	≥0	8,164 (97.7%)	5,165 (97.8%)	9,517 (98.1%)	14,778 (98.2%)
Postop EQ Utility Category	<0 ‘worse than dead’	36 (0.5%)	15 (0.3%)	28 (0.4%)	34 (0.3%)
	≥0	6,947 (99.5%)	4,513 (99.7%)	7,380 (99.6%)	13,052 (99.7%)
Patient Perceived Change	Improved	6,407 (92.5%)	4,117 (91.9%)	6,740 (91.8%)	11,976 (92.6%)
	Not-improved	522 (7.5%)	361 (8.1%)	599 (8.2%)	958 (7.4%)
Patient Satisfaction	Satisfied	5,630 (81.2%)	3,848 (85.9%)	6,299 (85.8%)	11,181 (86.4%)
	Not-satisfied	1,300 (18.8%)	630 (14.1%)	1,042 (14.2%)	1,757 (13.6%)
Responder	Responder	5,352 (82.6%)	3,413 (81.7%)	5,362 (81.2%)	9,069 (81%)
	Non-responder	1,130 (17.4%)	765 (18.3%)	1,245 (18.8%)	2,131 (19%)
TOTAL (TKA)		8,736	5,556	10,422	16,782
**REVERSE TOTAL SHOULDER ARTHROPLASTY (RTSA)**					
Preop EQ Utility Score	Mean ± SD	0.6 ± 0.3	0.7 ± 0.2	0.7 ± 0.2	0.7 ± 0.2
Preop EQ VAS Score	Mean ± SD	68.5 ± 19	70.4 ± 18.5	69.7 ± 18.9	70.9 ± 18
Preop OSS	Mean ± SD	22.1 ± 8.5	21.9 ± 9.3	23.3 ± 8.6	24.1 ± 9.1
Preop Joint Pain Score	Mean ± SD	7.4 ± 2	7.1 ± 2.2	7 ± 2	6.9 ± 2.2
Postop EQ Utility Score	Mean ± SD	0.9 ± 0.2	0.9 ± 0.2	0.9 ± 0.2	0.8 ± 0.2
Postop EQ VAS Score	Mean ± SD	76.3 ± 19.1	78.2 ± 15.1	77.7 ± 16.3	78.1 ± 17.2
Postop OSS	Mean ± SD	39.1 ± 7.2	38.5 ± 9.2	38.6 ± 7.9	39.4 ± 7.9
Postop Joint Pain Score	Mean ± SD	1.9 ± 2.2	2.2 ± 2.3	2.4 ± 2.5	2.2 ± 2.4
Preop EQ Utility Category	<0 ‘worse than dead’	4 (1.8%)	2 (1.9%)	5 (1.6%)	6 (1.2%)
	≥0	214 (98.2%)	105 (98.1%)	300 (98.4%)	512 (98.8%)
Postop EQ Utility Category	<0 ‘worse than dead’	2 (1.2%)		1 (0.4%)	3 (0.6%)
	≥0	164 (98.8%)	72 (100%)	241 (99.6%)	462 (99.4%)
Patient Perceived Change	Improved	157 (95.2%)	68 (94.4%)	217 (90.8%)	427 (93%)
	Not-improved	8 (4.8%)	4 (5.6%)	22 (9.2%)	32 (7%)
Patient Satisfaction	Satisfied	138 (83.6%)	62 (86.1%)	212 (88.7%)	403 (87.8%)
	Not-satisfied	27 (16.4%)	10 (13.9%)	27 (11.3%)	56 (12.2%)
Stemmed Reverse Shoulder Responder	Responder	125 (82.2%)	48 (78.7%)	167 (75.9%)	289 (75.9%)
	Non-responder	27 (17.8%)	13 (21.3%)	53 (24.1%)	92 (24.1%)
TOTAL (RTSA)		229	119	325	597

*SD* standard deviation, *OHS* oxford hip score, *OKS* oxford knee score, *OSS* oxford shoulder score, *VAS* visual analogue scale

Postoperatively, at least 90% of THA, TKA and RTSA patients reported they had improved following surgery, and at least 81% of patients were satisfied with the outcome (Table 2).

### Primary outcome – EQ-5D-5L

Pandemic year was significantly associated with preoperative and postoperative EQ-5D Utility scores for THA and TKA (*p* ≤ 0.001), but not RTSA (Tables 3, 4, 5, 6, 7 and 8). Quality of life reduced during the pandemic for THA and TKA; however, these decreases were very small (adjusted mean difference for pairwise comparisons ≤ 0.03 points) and well below the MCID. Pandemic year was also significantly associated with quality of life worse than dead, but only for preoperative THA. For people awaiting THA, the odds of having preoperative quality of life worse than dead increased by up to 40% during the pandemic years (ORs 1.24 to 1.40, *p* < 0.02).

**Table 3:** Total hip arthroplasty—estimated main effects of pandemic year on continuous outcomes

Outcome	*N*	Exposure or PW Comparison	*P* unadjusted model	Unadjusted MD (95% CI)	*P* adjusted model	Adjusted MD (95% CI)
Preop EQ Utility Score	24,517	Pandemic Year	<.0001		<.0001	
		Pandemic year 1 vs Pre-pandemic		−0.02 (−0.03, −0.01)		−0.03 (−0.04, −0.02)
		Pandemic year 2 vs Pre-pandemic		0.01 (0.00, 0.03)		−0.03 (−0.04, −0.02)
		Pandemic year 3 vs Pre-pandemic		0.01 (0.00, 0.02)		−0.03 (−0.04, −0.02)
Postop EQ Utility Score	20,669	Pandemic Year	<.0001		<.0001	
		Pandemic year 1 vs Pre-pandemic		−0.02 (−0.02, −0.01)		−0.02 (−0.03, −0.01)
		Pandemic year 2 vs Pre-pandemic		0.00 (−0.01, 0.01)		−0.02 (−0.02, −0.01)
		Pandemic year 3 vs Pre-pandemic		0.00 (−0.00, 0.01)		−0.01 (−0.02, −0.01)
Preop EQ VAS Score	24,245	Pandemic Year	0.0101		<.0001	
		Pandemic year 1 vs Pre-pandemic		−1.07 (−1.95, −0.20)		−1.26 (−2.11, −0.42)
		Pandemic year 2 vs Pre-pandemic		−0.72 (−1.46, 0.01)		−2.12 (−2.84, −1.40)
		Pandemic year 3 vs Pre-pandemic		−1.11 (−1.79, −0.44)		−2.67 (−3.34, −1.99)
Postop EQ VAS Score	20,489	Pandemic Year	<.0001		0.0001	
		Pandemic year 1 vs Pre-pandemic		−1.36 (−2.10, −0.62)		−1.51 (−2.23, −0.79)
		Pandemic year 2 vs Pre-pandemic		0.02 (−0.62, 0.66)		−1.17 (−1.80, −0.53)
		Pandemic year 3 vs Pre-pandemic		0.36 (−0.21, 0.93)		−0.79 (−1.37, −0.21)
Preop Joint Pain Score	24,208	Pandemic Year	<.0001		0.0002	
		Pandemic year 1 vs Pre-pandemic		0.16 (0.07, 0.25)		0.19 (0.10, 0.28)
		Pandemic year 2 vs Pre-pandemic		−0.10 (−0.18, −0.02)		0.08 (0.01, 0.16)
		Pandemic year 3 vs Pre-pandemic		−0.09 (−0.16, −0.02)		0.11 (0.04, 0.18)
Postop Joint Pain Score	20,494	Pandemic Year	0.0005		<.0001	
		Pandemic year 1 vs Pre-pandemic		0.21 (0.10, 0.31)		0.23 (0.12, 0.33)
		Pandemic year 2 vs Pre-pandemic		0.02 (−0.06, 0.11)		0.16 (0.07, 0.25)
		Pandemic year 3 vs Pre-pandemic		0.08 (−0.00, 0.16)		0.21 (0.13, 0.29)
Preop OHS Score	24,253	Pandemic Year	<.0001		<.0001	
		Pandemic year 1 vs Pre-pandemic		−0.42 (−0.82, −0.03)		−0.62 (−0.99, −0.26)
		Pandemic year 2 vs Pre-pandemic		0.67 (0.34, 1.00)		−0.54 (−0.85, −0.23)
		Pandemic year 3 vs Pre-pandemic		0.62 (0.32, 0.93)		−0.68 (−0.97, −0.39)
Postop OHS Score	20,524	Pandemic Year	<.0001		<.0001	
		Pandemic year 1 vs Pre-pandemic		−0.92 (−1.25, −0.58)		−1.03 (−1.36, −0.71)
		Pandemic year 2 vs Pre-pandemic		0.03 (−0.26, 0.32)		−0.76 (−1.05, −0.48)
		Pandemic year 3 vs Pre-pandemic		0.09 (−0.17, 0.35)		−0.68 (−0.94, −0.42)

The crude model adjusted for pandemic year (pre-pandemic/pandemic year 1/pandemic year 2/pandemic year 3). The adjusted model adjusted for pandemic year (pre-pandemic/pandemic year 1/pandemic year 2/pandemic year 3), age group (< 65/>  = 65 years), gender (female/male), ASA score (1/2/3/4–5), BMI category (under or normal weight/pre-obese/obese class 1/obese class 2/obese class 3), state (VIC/Other states or territories), hospital system (private/public). *PROM* patient reported outcome measure, *MD* mean difference, *OR* odds ratio, *CI* confidence interval, *PW* pair wise

**Table 4:** Total hip arthroplasty—estimated main effects of pandemic year on binary outcomes

Outcome	*N*	Exposure or PW Comparison	*P* unadjusted model	Unadjusted OR (95% CI)	*P* adjusted model	Adjusted OR (95% CI)
Preop EQ Utility Category	24,517	Pandemic Year	0.0038		0.0013	
		Pandemic year 1 vs Pre-pandemic		1.22 (1.01, 1.48)		1.30 (1.07, 1.58)
		Pandemic year 2 vs Pre-pandemic		0.86 (0.72, 1.03)		1.24 (1.04, 1.49)
		Pandemic year 3 vs Pre-pandemic		0.94 (0.80, 1.11)		1.40 (1.18, 1.65)
Postop EQ Utility Category	20,669	Pandemic Year	0.7099		0.1512	
		Pandemic year 1 vs Pre-pandemic		1.19 (0.61, 2.32)		1.20 (0.61, 2.35)
		Pandemic year 2 vs Pre-pandemic		0.86 (0.46, 1.61)		1.29 (0.68, 2.44)
		Pandemic year 3 vs Pre-pandemic		1.15 (0.68, 1.96)		1.83 (1.05, 3.18)
Patient Perceived Change	20,473	Pandemic Year	0.0101		0.0270	
		Pandemic year 1 vs Pre-pandemic		0.74 (0.58, 0.95)		0.72 (0.56, 0.93)
		Pandemic year 2 vs Pre-pandemic		0.96 (0.76, 1.20)		0.87 (0.69, 1.10)
		Pandemic year 3 vs Pre-pandemic		1.08 (0.87, 1.33)		0.99 (0.79, 1.23)
Patient Satisfaction	20,478	Pandemic Year	<.0001		<.0001	
		Pandemic year 1 vs Pre-pandemic		1.53 (1.31, 1.78)		1.51 (1.29, 1.76)
		Pandemic year 2 vs Pre-pandemic		2.05 (1.78, 2.36)		1.84 (1.59, 2.13)
		Pandemic year 3 vs Pre-pandemic		2.14 (1.89, 2.42)		1.91 (1.68, 2.18)
Responder	18,494	Pandemic Year	0.2194		0.1873	
		Pandemic year 1 vs Pre-pandemic		0.91 (0.77, 1.07)		0.90 (0.77, 1.06)
		Pandemic year 2 vs Pre-pandemic		0.91 (0.79, 1.05)		0.90 (0.78, 1.04)
		Pandemic year 3 vs Pre-pandemic		0.87 (0.76, 0.99)		0.86 (0.75, 0.98)

The crude model adjusted for pandemic year (pre-pandemic/pandemic year 1/pandemic year 2/pandemic year 3). The adjusted model adjusted for pandemic year (pre-pandemic/pandemic year 1/pandemic year 2/pandemic year 3), age group (< 65/>  = 65 years), gender (female/male), ASA score (1/2/3/4–5), BMI category (under or normal weight/pre-obese/obese class 1/obese class 2/obese class 3), state (VIC/Other states or territories), hospital system (private/public)

*PROM* patient reported outcome measure, *MD* mean difference, *OR* odds ratio, *CI* confidence interval, *PW* pair wise

**Table 5:** Total knee arthroplasty—estimated main effects of pandemic on continuous outcomes

Outcome	*N*	Exposure or PW Comparison	*P*unadjusted model	Unadjusted MD (95% CI)	*P*adjusted model	Adjusted MD (95% CI)
Preop EQ Utility Score	38,108	Pandemic Year	<.0001		0.0015	
		Pandemic year 1 vs Pre-pandemic		−0.00 (−0.01, 0.01)		−0.01 (−0.02, 0.00)
		Pandemic year 2 vs Pre-pandemic		0.02 (0.01, 0.03)		−0.01 (−0.02, −0.00)
		Pandemic year 3 vs Pre-pandemic		0.02 (0.01, 0.03)		−0.01 (−0.02, −0.01)
Postop EQ Utility Score	31,787	Pandemic Year	0.0092		<.0001	
		Pandemic year 1 vs Pre-pandemic		−0.00 (−0.01, 0.00)		−0.01 (−0.01, 0.00)
		Pandemic year 2 vs Pre-pandemic		0.00 (−0.00, 0.01)		−0.01 (−0.02, −0.01)
		Pandemic year 3 vs Pre-pandemic		0.01 (0.00, 0.01)		−0.01 (−0.02, −0.01)
Preop EQ VAS Score	37,661	Pandemic Year	<.0001		0.0136	
		Pandemic year 1 vs Pre-pandemic		0.77 (0.13, 1.40)		0.48 (−0.13, 1.09)
		Pandemic year 2 vs Pre-pandemic		1.12 (0.58, 1.66)		−0.27 (−0.80, 0.26)
		Pandemic year 3 vs Pre-pandemic		1.16 (0.67, 1.66)		−0.43 (−0.92, 0.06)
Postop EQ VAS Score	31,443	Pandemic Year	<.0001		0.0149	
		Pandemic year 1 vs Pre-pandemic		−0.34 (−0.93, 0.24)		−0.55 (−1.12, 0.01)
		Pandemic year 2 vs Pre-pandemic		0.66 (0.15, 1.17)		−0.63 (−1.14, −0.13)
		Pandemic year 3 vs Pre-pandemic		0.71 (0.26, 1.16)		−0.74 (−1.19, −0.28)
Preop Joint Pain Score	37,527	Pandemic Year	<.0001		0.6666	
		Pandemic year 1 vs Pre-pandemic		−0.02 (−0.09, 0.06)		0.03 (−0.04, 0.10)
		Pandemic year 2 vs Pre-pandemic		−0.16 (−0.22, −0.10)		0.02 (−0.04, 0.08)
		Pandemic year 3 vs Pre-pandemic		−0.18 (−0.24, −0.12)		0.03 (−0.02, 0.09)
Postop Joint Pain Score	31,483	Pandemic Year	0.0602		<.0001	
		Pandemic year 1 vs Pre-pandemic		0.11 (0.02, 0.20)		0.14 (0.05, 0.23)
		Pandemic year 2 vs Pre-pandemic		0.07 (−0.01, 0.15)		0.23 (0.15, 0.31)
		Pandemic year 3 vs Pre-pandemic		0.08 (0.01, 0.15)		0.26 (0.19, 0.34)
Preop OKS Score	37,587	Pandemic Year	<.0001		0.3604	
		Pandemic year 1 vs Pre-pandemic		0.28 (−0.01, 0.57)		0.01 (−0.26, 0.28)
		Pandemic year 2 vs Pre-pandemic		0.93 (0.69, 1.18)		−0.18 (−0.42, 0.05)
		Pandemic year 3 vs Pre-pandemic		1.23 (1.00, 1.45)		−0.06 (−0.28, 0.16)
Postop OKS Score	31,524	Pandemic Year	<.0001		<.0001	
		Pandemic year 1 vs Pre-pandemic		−0.14 (−0.44, 0.15)		−0.27 (−0.55, 0.02)
		Pandemic year 2 vs Pre-pandemic		0.18 (−0.07, 0.44)		−0.57 (−0.83, −0.32)
		Pandemic year 3 vs Pre-pandemic		0.41 (0.19, 0.64)		−0.44 (−0.67, −0.21)

The crude model adjusted for pandemic year (pre-pandemic/pandemic year 1/pandemic year 2/pandemic year 3). The adjusted model adjusted for pandemic year (pre-pandemic/pandemic year 1/pandemic year 2/pandemic year 3), age group (< 65/>  = 65 years), gender (female/male), ASA score (1/2/3/4–5), BMI category (under or normal weight/pre-obese/obese class 1/obese class 2/obese class 3), state (VIC/Other states or territories), hospital system (private/public)

*PROM* patient reported outcome measure, *MD* mean difference, *OR* odds ratio, *CI* confidence interval, *PW* pair wise

**Table 6:** Total knee arthroplasty—estimated main effects of pandemic year on binary outcomes

Outcomes	*N*	Exposure or PW Comparison	*P* unadjusted model	Unadjusted OR (95% CI)	*P* adjusted model	Adjusted OR (95% CI)
Preop EQ Utility Category	38,108	Pandemic Year	0.1073		0.2016	
		Pandemic year 1 vs Pre-pandemic		0.97 (0.77, 1.23)		1.05 (0.82, 1.33)
		Pandemic year 2 vs Pre-pandemic		0.83 (0.68, 1.02)		1.19 (0.96, 1.47)
		Pandemic year 3 vs Pre-pandemic		0.82 (0.68, 0.99)		1.21 (1.00, 1.48)
Postop EQ Utility Category	31,787	Pandemic Year	0.0536		0.4777	
		Pandemic year 1 vs Pre-pandemic		0.66 (0.36, 1.21)		0.69 (0.37, 1.26)
		Pandemic year 2 vs Pre-pandemic		0.70 (0.42, 1.16)		0.97 (0.58, 1.64)
		Pandemic year 3 vs Pre-pandemic		0.52 (0.32, 0.83)		0.75 (0.46, 1.23)
Patient Perceived Change	31,466	Pandemic Year	0.2168		0.0387	
		Pandemic year 1 vs Pre-pandemic		0.93 (0.81, 1.07)		0.92 (0.80, 1.06)
		Pandemic year 2 vs Pre-pandemic		0.91 (0.81, 1.03)		0.83 (0.73, 0.94)
		Pandemic year 3 vs Pre-pandemic		1.01 (0.90, 1.13)		0.90 (0.80, 1.01)
Patient Satisfaction	31,473	Pandemic Year	<.0001		<.0001	
		Pandemic year 1 vs Pre-pandemic		1.41 (1.27, 1.57)		1.40 (1.26, 1.55)
		Pandemic year 2 vs Pre-pandemic		1.39 (1.27, 1.52)		1.28 (1.17, 1.41)
		Pandemic year 3 vs Pre-pandemic		1.47 (1.36, 1.59)		1.33 (1.22, 1.44)
Responder	28,279	Pandemic Year	0.0568		0.1212	
		Pandemic year 1 vs Pre-pandemic		0.94 (0.85, 1.04)		0.95 (0.86, 1.05)
		Pandemic year 2 vs Pre-pandemic		0.91 (0.83, 0.99)		0.92 (0.84, 1.01)
		Pandemic year 3 vs Pre-pandemic		0.90 (0.83, 0.97)		0.91 (0.83, 0.98)

The crude model adjusted for pandemic year (pre-pandemic/pandemic year 1/pandemic year 2/pandemic year 3). The adjusted model adjusted for pandemic year (pre-pandemic/pandemic year 1/pandemic year 2/pandemic year 3), age group (< 65/>  = 65 years), gender (female/male), ASA score (1/2/3/4–5), BMI category (under or normal weight/pre-obese/obese class 1/obese class 2/obese class 3), state (VIC/Other states or territories), hospital system (private/public)

*PROM* patient reported outcome measure, *MD* mean difference, *OR* odds ratio, *CI* confidence interval, *PW* pair wise

**Table 7:** Reverse total shoulder arthroplasty—estimated main effects of pandemic year on continuous outcomes

Outcome	*N*	Exposure or PW Comparison	*P* unadjusted model	Unadjusted MD (95% CI)	*P* adjusted model	Adjusted MD (95% CI)
Preop EQ Utility Score	1136	Pandemic Year	0.0039		0.2235	
		Pandemic year 1 vs Pre-pandemic		0.02 (−0.04, 0.08)		0.02 (−0.03, 0.08)
		Pandemic year 2 vs Pre-pandemic		0.06 (0.02, 0.10)		0.04 (0.00, 0.08)
		Pandemic year 3 vs Pre-pandemic		0.07 (0.03, 0.11)		0.03 (−0.01, 0.07)
Postop EQ Utility Score	936	Pandemic Year	0.7106		0.3816	
		Pandemic year 1 vs Pre-pandemic		0.00 (−0.05, 0.06)		−0.01 (−0.06, 0.04)
		Pandemic year 2 vs Pre-pandemic		−0.01 (−0.05, 0.03)		−0.02 (−0.06, 0.02)
		Pandemic year 3 vs Pre-pandemic		−0.02 (−0.05, 0.02)		−0.03 (−0.06, 0.00)
Preop EQ VAS Score	1119	Pandemic Year	0.4355		0.8646	
		Pandemic year 1 vs Pre-pandemic		1.70 (−2.62, 6.02)		1.71 (−2.42, 5.85)
		Pandemic year 2 vs Pre-pandemic		1.20 (−2.06, 4.46)		0.21 (−2.95, 3.36)
		Pandemic year 3 vs Pre-pandemic		2.43 (−0.55, 5.41)		0.52 (−2.41, 3.44)
Postop EQ VAS Score	926	Pandemic Year	0.8033		0.9866	
		Pandemic year 1 vs Pre-pandemic		1.61 (−3.19, 6.40)		0.70 (−3.93, 5.33)
		Pandemic year 2 vs Pre-pandemic		1.20 (−2.23, 4.64)		0.54 (−2.76, 3.85)
		Pandemic year 3 vs Pre-pandemic		1.53 (−1.56, 4.61)		0.47 (−2.54, 3.48)
Preop Joint Pain Score	1121	Pandemic Year	0.0200		0.1446	
		Pandemic year 1 vs Pre-pandemic		−0.37 (−0.86, 0.13)		−0.36 (−0.84, 0.11)
		Pandemic year 2 vs Pre-pandemic		−0.38 (−0.75, −0.01)		−0.33 (−0.69, 0.03)
		Pandemic year 3 vs Pre-pandemic		−0.54 (−0.88, −0.20)		−0.39 (−0.72, −0.05)
Postop Joint Pain Score	927	Pandemic Year	0.4070		0.2628	
		Pandemic year 1 vs Pre-pandemic		0.21 (−0.45, 0.87)		0.33 (−0.32, 0.99)
		Pandemic year 2 vs Pre-pandemic		0.41 (−0.06, 0.89)		0.46 (−0.01, 0.94)
		Pandemic year 3 vs Pre-pandemic		0.24 (−0.18, 0.67)		0.37 (−0.06, 0.79)
Preop OSS Score	1123	Pandemic Year	0.0148		0.7943	
		Pandemic year 1 vs Pre-pandemic		0.04 (−2.03, 2.11)		0.20 (−1.69, 2.09)
		Pandemic year 2 vs Pre-pandemic		1.33 (−0.23, 2.90)		0.59 (−0.85, 2.04)
		Pandemic year 3 vs Pre-pandemic		2.07 (0.64, 3.50)		0.63 (−0.70, 1.96)
Postop OSS Score	928	Pandemic Year	0.5775		0.7168	
		Pandemic year 1 vs Pre-pandemic		−0.53 (−2.71, 1.66)		−1.07 (−3.21, 1.08)
		Pandemic year 2 vs Pre-pandemic		−0.34 (−1.91, 1.22)		−0.61 (−2.14, 0.93)
		Pandemic year 3 vs Pre-pandemic		0.42 (−0.99, 1.83)		−0.22 (−1.62, 1.18)

The crude model adjusted for pandemic year (pre-pandemic/pandemic year 1/pandemic year 2/pandemic year 3). The adjusted model adjusted for pandemic year (pre-pandemic/pandemic year 1/pandemic year 2/pandemic year 3), age group (< 65/>  = 65 years), gender (female/male), ASA score (1/2/3/4–5), BMI category (under or normal weight/pre-obese/obese class 1/obese class 2/obese class 3), state (VIC/Other states or territories), hospital system (private/public)

*PROM* patient reported outcome measure, *MD* mean difference, *OR* odds ratio, *CI* confidence interval, *PW* pair wise

**Table 8:** Reverse total shoulder arthroplasty—estimated main effects of pandemic year on binary outcomes

Outcome	*N*	Exposure or PW Comparison	*P* unadjusted model	Unadjusted OR (95% CI)	*P* adjusted model	Adjusted OR (95% CI)
Preop EQ Utility Category	1136	Pandemic Year	0.8683		0.9456	
		Pandemic year 1 vs Pre-pandemic		1.01 (0.18, 5.60)		0.80 (0.14, 4.68)
		Pandemic year 2 vs Pre-pandemic		0.88 (0.23, 3.32)		1.27 (0.31, 5.19)
		Pandemic year 3 vs Pre-pandemic		0.62 (0.17, 2.22)		1.26 (0.33, 4.84)
Patient Perceived Change	926	Pandemic Year	0.3834		0.3217	
		Pandemic year 1 vs Pre-pandemic		0.88 (0.26, 3.03)		0.70 (0.20, 2.44)
		Pandemic year 2 vs Pre-pandemic		0.51 (0.22, 1.17)		0.45 (0.19, 1.06)
		Pandemic year 3 vs Pre-pandemic		0.69 (0.31, 1.52)		0.57 (0.25, 1.29)
Patient Satisfaction	926	Pandemic Year	0.4176		0.5466	
		Pandemic year 1 vs Pre-pandemic		1.24 (0.57, 2.72)		1.07 (0.48, 2.38)
		Pandemic year 2 vs Pre-pandemic		1.56 (0.87, 2.77)		1.49 (0.83, 2.67)
		Pandemic year 3 vs Pre-pandemic		1.45 (0.88, 2.40)		1.32 (0.79, 2.21)
Responder	806	Pandemic Year	0.4931		0.5840	
		Pandemic year 1 vs Pre-pandemic		0.82 (0.39, 1.71)		0.79 (0.37, 1.68)
		Pandemic year 2 vs Pre-pandemic		0.71 (0.42, 1.19)		0.71 (0.41, 1.21)
		Pandemic year 3 vs Pre-pandemic		0.70 (0.43, 1.13)		0.72 (0.44, 1.18)

The crude model adjusted for pandemic year (pre-pandemic/pandemic year 1/pandemic year 2/pandemic year 3). The adjusted model adjusted for pandemic year (pre-pandemic/pandemic year 1/pandemic year 2/pandemic year 3), age group (< 65/>  = 65 years), gender (female/male), ASA score (1/2/3/4–5), BMI category (under or normal weight/pre-obese/obese class 1/obese class 2/obese class 3), state (VIC/Other states or territories), hospital system (private/public)

Postop EQ Utility Category not analysed due to low number of positive cases

*PROM* patient reported outcome measure, *MD* mean difference, *OR* odds ratio, *CI* confidence interval, *PW* pair wise

### Hip arthroplasty—secondary outcomes and covariates

For THA, the pandemic year was associated with 8 of the 9 secondary outcomes after adjusting for other covariates (*p* ≤ 0.0001, Tables 3 and 4). Significant mean differences for pairwise comparisons (pre-pandemic compared to pandemic) were small and well below MCIDs. The likelihood of being satisfied with surgery was 4.1 to 6.4 percentage points higher during the pandemic than pre-pandemic (ORs 1.51 to 1.91, *p* < 0.001).

Age and gender covariates did not modify the effects of pandemic year on outcomes. Interactions between the hospital system (public versus private hospital) and pandemic year were inconsistent across the 13 outcomes tested; six of the 13 outcomes had significant interactions (*p* < 0.05).

### Knee arthroplasty—secondary outcomes and covariates

For TKA, the pandemic year was associated with six of the nine secondary outcomes after adjusting for other covariates (*p* < 0.05, Tables 5 and 6). Significant mean differences for pairwise comparisons (pre-pandemic compared to pandemic) were small and well below MCIDs.

The likelihood of being satisfied with surgery was 4.6 to 5.2 percentage points higher during the pandemic than pre-pandemic (ORs 1.28 to 1.40, *p* < 0.0001).

Regarding covariates, age, gender and hospital system did not modify the effects of pandemic year for most TKA outcomes.

### Shoulder arthroplasty—secondary outcomes and covariates

For patients undergoing RTSA, the pandemic year was not significantly associated with any outcome after adjusting for covariates (*p* > 0.05, Tables 7 and 8). No significant differences were observed for pairwise comparisons between the pre-pandemic period and each pandemic year for all but two of the 39 comparisons.

Age, gender and hospital system were not significantly associated with any outcome (*p* > 0.05).

## Discussion

Using national-level data for patients undergoing THA, TKA and RTSA in Australia, we found little change in preoperative and postoperative quality of life and other patient-reported outcomes during the pandemic compared to the pre-pandemic period. While there was an increase in the proportion of people awaiting THA with quality of life worse than dead during the pandemic, this increase was relatively modest and not evident for the TKA or RTSA cohorts. Compared to before the pandemic, patient satisfaction increased modestly during the pandemic for THA and TKA.

Advanced joint disease produces substantial declines in quality of life, and the COVID-19 pandemic could further deteriorate quality of life for a range of reasons, including lengthening waiting times for arthroplasty [5]. With the exception of a modest increase in the proportion of patients awaiting THA with a quality of life worse than dead, our results indicated that preoperative quality of life remained fairly stable during the pandemic for people awaiting surgery. Prior to the pandemic, Scott et al. (2019) reported that 19% and 12% of patients awaiting THA and TKA, respectively, had quality of life worse than dead at a single health service in the UK [4]. During the pandemic, Clement et al. (2021) reported that 35% and 22% of hip and knee arthroplasty patients had quality of life worse than dead, implying the pandemic had contributed to declining quality of life [5]. However, these studies included different health services, so direct comparisons cannot be made. National data from the UK’s National Joint Registry [18] indicated mean preoperative EQ-5D Utility Scores of 0.33 and 0.43 for hips and knees, respectively, which is substantially higher than the scores reported by Clement et al. (hips = 0.24, knees = 0.35), yet similar to those reported by Scott et al. (hips = 0.36, knees 0.41). Given that patients in Clement et al.’s study had poorer preoperative quality of life than the national average, this cautions against generalising their findings to other jurisdictions. In our study, mean preoperative EQ-5D Utility Scores were typically 0.20 to 0.30 points higher than UK averages. These differences between countries likely reflect different preferences for the various health states quantified by the EQ-5D Utility Score. Preferences vary between countries and can change over time. In Australia, the EQ-5D value set was recently updated using contemporary preferencing methods and a four times larger sample [9]. When applied to Australian patients awaiting arthroplasty, the new value set produced mean preoperative EQ-5D Utility Scores that were 0.17 to 0.21 units higher than the previous value set, and fewer patients had quality of life worse than dead [19]. In the present study, preoperative Oxford Scores were similar to those reported by Scott et al. suggesting similar pain levels and functional status and further reinforcing the need to carefully interpret EQ-5D Utility Scores based on differences in country-specific value sets.

For THA patients, the odds of having preoperative quality of life worse than dead increased by up to 40% during the pandemic. This effect was not apparent for the TKA and RTSA cohorts. Scott et al. [4]. and Clement et al. [5]. reported that the proportion of patients with quality of life worse than dead was at least 50% higher in hips than in knees. Each joint has distinct pathogenic processes. Hip OA can progress rapidly from mild to severe pain [20], and these people opt for arthroplasty sooner after the onset of symptoms, which might explain the higher proportion of worse than dead patients awaiting THA.

Satisfaction with the outcomes of THA and TKA changed over time, with a small increase in the proportion of patients being satisfied during the pandemic than before (approximately 5% points, ORs 1.3 to 1.9). This effect was modified by the hospital system. Patients treated in private hospitals were more likely to be satisfied with surgery than patients treated in public hospitals; this effect was significant during the pandemic but not before. The reasons for this effect are speculative. Satisfaction with arthroplasty is influenced by multiple factors, including meeting patient expectations and the quality of the patient’s hospital experience [21]. It is possible that differences between private and public patients existed for these factors, creating differences in satisfaction rates in our study.

We acknowledge the limitations of the current study. Causal attributions are not possible, and any changes that were temporally associated with the pandemic cannot be definitely attributed to the pandemic. Other factors could have contributed, and we used a cohort study design to adjust for potential covariates using available data. To mitigate the potential risk of Type 1 errors from multiple comparisons, we required consistency in outcomes across several measures before considering an effect present, and we also considered the significance of the findings with regard to effect size and clinical importance. MCIDs have their own limitations and were not available from the literature for all measures [22]. The shoulder cohort was relatively small, which reduced statistical power and the likelihood of finding significant differences between groups. Nonetheless, confidence intervals were small and clinical important differences remain unlikely even with a larger sample. Selection bias might have influenced results. Patients being offered and receiving surgery during the pandemic might have had different characteristics to patients undergoing surgery before the pandemic. Although our results indicated that patients were similar for a limited number of preoperative characteristics (age, gender, BMI, ASA, symptom severity), unmeasured characteristics might have been different between the groups. At a national level, the number of patients undergoing arthroplasty declined rapidly, particularly in the early stages of the pandemic, however, we did not measure changes in waiting time for surgery, and its influence on outcomes remains unknown. The AOANJRR PROMs program included approximately 74% of patients preoperatively and 62% postoperatively [6], hence, missing data creates uncertainty regarding the generalisability of the results to all patients undergoing arthroplasty. Generalisability of the findings beyond Australia is also uncertain, given differences in health systems and EQ-5D value sets. The EQ-5D-5L is generic health-related quality of life instrument, and together with our joint-specific instruments such as the Oxford scores, might have been insensitive to detecting COVID-related changes in quality of life. Results are limited to people with the primary diagnosis of OA, although this is the most common indication for hip, knee and shoulder arthroplasty in Australia [6].

## Conclusion

Using a large national dataset, the current study demonstrated that the COVID-19 pandemic was not associated with a clinically meaningful deterioration in pre- or postoperative quality of life for patients undergoing THA, TKA or RTSA in Australia. There was a small increase in the (relatively low) proportion of THA patients who experienced preoperative quality of life worse than dead during the pandemic than before, but this difference was not seen postoperatively or in TKA or RTSA patients. Overall, quality of life in patients undergoing joint replacement surgery in Australia was resilient during the COVID-19 pandemic.

## Data Availability

The data that support the findings of this study were obtained from the Australian Orthopaedic Association National Joint Replacement Registry (AOANJRR). Access to these data is governed by AOANJRR data access and release policies, and the data were provided under licence for the purposes of the current study. As such, the data are not publicly available. Data may be made available from the authors upon reasonable request and subject to approval from the AOANJRR.

## Abbreviations

*AOANJRR*: Australian Orthopaedic Association National Joint Replacement Registry
*ASA*: American Society of Anesthesiologists
*BMI*: Body mass index
*CI*: Confidence interval
*EQ-5D-5L*: EuroQol 5-dimension, 5-level instrument
*EQ VAS*: EuroQol visual analogue scale
*MCIC*: Minimal clinically important change
*MCID*: Minimal clinically important difference
*MD*: Mean difference
*NJR*: National Joint Registry (UK)
*OA*: Osteoarthritis
*OHS*: Oxford hip score
*OKS*: Oxford knee score
*OR*: Odds ratio
*OSS*: Oxford shoulder score
*PROMs*: Patient-reported outcome measures
*PW*: Pairwise
*QOL*: Quality of life
*RTSA*: Reverse total shoulder arthroplasty
*SD*: Standard deviation
*TKA*: Total knee arthroplasty
*TSA*: Total shoulder arthroplasty
*THA*: Total hip arthroplasty
*VIC*: Victoria (Australian state)
*WTD*: Worse than dead
